# A cross-sectional investigation of the mental health and wellbeing among individuals who have been negatively impacted by the COVID-19 international border closure in Australia

**DOI:** 10.1186/s12992-022-00807-7

**Published:** 2022-02-08

**Authors:** Kathina Ali, Matthew Iasiello, Joep van Agteren, Teri Mavrangelos, Michael Kyrios, Daniel B. Fassnacht

**Affiliations:** 1 College of Education, Psychology and Social Work, Flinders University, Sturt Road, Bedford Park, South Australia 5042 Australia; 2 Órama Institute for Mental Health & Wellbeing, Flinders University, Sturt Road, Bedford Park, South Australia 5042 Australia; 3 Wellbeing and Resilience Centre, Lifelong Health Theme, South Australian Health and Medical Research Institute, North Terrace, Adelaide, South Australia 5000 Australia; 4 College of Nursing and Health Science, Flinders University, Sturt Road, Bedford Park, South Australia 5042 Australia

**Keywords:** COVID-19, Coronavirus, International border closures, Mental health, Psychological distress, Wellbeing

## Abstract

**Background:**

The COVID-19 pandemic resulted in the Australian government implementing strict international border closures. However, research has not yet investigated the mental health status of individuals impacted negatively by these international border closures.

**Methods:**

The present study was a cross-sectional online survey of 3968 adults who reported being negatively affected by the border closure during June and July 2021. Psychological distress was measured with the Kessler Psychological Distress Scale (K10), stress with the Perceived Stress Scale (PSS) and wellbeing with the Mental Health Continuum Short Form (MHC-SF).

**Results:**

In total, 3968 participants reported being negatively affected by the current restrictions (63.4% in Australia, 36.6% overseas). The vast majority of respondents (83.6%) reported high or very high levels of psychological distress (mean K10 score > 22), and 74.8% reported poor mental wellbeing, with similar risk profiles for participants in Australia or overseas. The most common scenarios of affected individuals included 1) wanting to enter Australia (30.8%), 2) wanting to leave Australia (29.6%) and 3) wanting someone to enter Australia (25.6%). Reasons included wanting to be with partners, family and friends (81.1%), for employment/economic reasons (4.9%), study (4.1%), personal safety/health (2.6%) or holiday (1.4%). While psychological distress was extremely high across all groups, separated partners and those with interrupted study experienced the highest distress (mean K10 = 35.7, ***n*** = 155).

**Conclusion:**

The data suggests a highly elevated mental health risk profile among individuals who report being negatively affected by current Australian international border closures. The results provide valuable data to inform future policy decisions and have clear implications regarding effective service provision for this vulnerable group.

**Supplementary Information:**

The online version contains supplementary material available at 10.1186/s12992-022-00807-7.

## Background

The onset of the COVID-19 pandemic has not only caused a significant challenge to the physical health but also to the mental health and wellbeing of individuals across the world. Mental health challenges are typically attributed to reasons beyond exposure to (or the direct fear of contracting) the virus [1, 2], particularly in Australia where the occurrence of infections has been comparatively attenuated [3] as a result of strict mitigation measures. In this context, mental health challenges can encompass elevated levels of psychological distress and/or low levels of wellbeing [4]. COVID-19 mental health consequences are often linked to direct and indirect implications from community restrictions and lockdowns, and their flow-on effects such as financial distress, social and/or work impairment [5–7], and decreased social connections and feelings of loneliness [8, 9]. While the mental health impact of common restrictions such as lockdowns is increasingly being studied around the globe (e.g., [10]), less attention has been paid to the specific impact or consequences of strict border closures [11].

At the beginning of the COVID-19 pandemic, the Australian government closed its international borders to reduce the risk of contracting and spreading the virus in Australia [12]. Since then, travel to or from Australia is only available if individuals are exempt or have been granted an individual exemption from the Commissioner of the Australian Border Force [13]. Although Australian citizens, permanent residents and their immediate family (i.e., spouses, de facto partner, dependent child, legal guardians and dependents) are technically exempt from these restrictions, many are experiencing difficulties entering Australia due to weekly arrival caps on the number of individuals allowed entry into the country which were further reduced, bringing the total number of weekly state and territory intake to around 3000 individuals (as of June 2021) [14]. For others, including parents (who are not considered immediate family members) and visa holders it is extremely difficult to enter the country as they require individual travel exemptions, for example, for compassionate and compelling reasons. However, these are commonly rejected with data from March 2020 to September 2020 indicating that from 53,212 applications received more than 90% were rejected [15]. In addition, Australia is one of the few countries which requires citizens, including dual citizens and permanent residents to obtain an exemption to leave the country which are often rejected as well. While 29% of countries worldwide have their borders closed for international travel (as of June 2021) [16], Australia is one of the few countries with such strict inbound and outbound travel restrictions. Given that almost 30% of Australians were born overseas, up to 50% were either born overseas or have at least one parent born overseas [17], and the fact that many Australians currently overseas are attempting to return home, the international border closure policy has the potential to impact significantly on the mental health of a sizeable proportion of the population. In fact, a recent scoping review has identified a variety of harmful unintended outcomes, including wellbeing and mental health, of international travel measures during the COVID-19 pandemic [11]. However, empirical data on important characteristics, for example, the mental health and wellbeing of those directly impacted by the current Australian border restrictions, has not yet been established.

Gaining an understanding of the mental health status for those who are directly affected by the international border closure is crucial to shape current and future policy decisions as well as the service provision and care of those affected by policies. In this article, we report cross-sectional data on the mental health and wellbeing of a sample of individuals who self-reported being negatively affected by the current international border closure. The specific aims were to investigate levels of psychological distress, perceived stress and mental wellbeing among this self-selected sample and subsequently use this data to identify particularly vulnerable groups for future studies.

## Method

The present study was a cross-sectional online survey with the objective to investigate the mental health status of individuals impacted negatively by the Australian COVID-19 international border closures.

### Participants and procedure

Participants were recruited via traditional (radio, TV) and social media including Facebook posts in relevant groups (e.g., “Travel Exemption Australia”, “Australians Stuck Around the World”, “Parents are Immediate Family Members”) and paid Facebook ads targeting individuals who may have been affected by the current COVID-19 Australian international border closure. Interested participants completed a 30-min anonymous online survey from 22 June until 27 July 2021. There were no specific inclusion criteria to participate in the study.

### Measures

#### Socio-demographic characteristics

Socio-demographic variables included age, gender, education, citizenship, current location, state (if in Australia), country of birth, ethnicity, education, employment, income, marital status, and children.

#### Impact of the COVID-19 international border closure (scenario and reason)

We asked participants whether and to what degree the restrictions had negatively affected their life or life plans (positively/negatively affected, visual analogue scale, 0 – 100). We asked participants to self-identify which of the following 6 scenarios best described their current situation. While participants may have been affected in different ways, they were specifically asked to only select the reason that had mostly affected them. We asked participants residing in Australia whether they were affected because they 1) wanted to leave Australia, 2) wanted someone to come to Australia or 3) other (a text box was provided where participants could elaborate on their experience) and participants overseas whether they were affected because they 4) wanted to enter Australia, 5) wanted someone to leave from Australia or 3) other. We further asked participants to indicate the main reason why they were affected by selecting one of the following options: separation from partner/family/friends, employment, study, personal safety/health, holiday, or other. We then asked to what extent they perceived this specific reason had negatively affected their mental health (visual analogue scale, 0 – 100).

#### Mental health and wellbeing

Psychological distress was assessed with the Kessler Psychological Distress Scale (K10) allowing for comparison with the Australian general population. The 10 items are rated on a five-point Likert scale (ranging from “none of the time” to “all of the time”) resulting in a score of 10 to 50, with greater scores indicating greater levels of psychological distress (range 10 – 50, 10-15: low, 16-21: moderate, 22-29: high, 30-50: very high) [18].

Stress was measured with the Perceived Stress Scale (PSS) which assesses perception of stress. Eight items of the PSS were selected for use, based on a recent validation of the PSS in an Australian sample [19]. The items are rated on a five-point Likert scale (ranging from “never” to “very often”). Items 1-3, 11, and 14 were used to score the Perceived Stress subscale (score of 0 to 25), while items 6, 7, 10 were used to score the Perceived Control subscale (score of 0 to 15) [19], with greater scores indicating greater levels of perceived stress.

Wellbeing was assessed with the Mental Health Continuum Short Form (MHC-SF). The 14 items measure emotional (hedonic), psychological (eudaimonic) and social wellbeing, and are rated on a six-point Likert scale (ranging from “never” to “every day”) resulting in a score of 0 to 70, with greater scores indicating greater levels of mental wellbeing [20]. The MHC-SF has been validated and allows categorization of individual’s functioning as (a) “flourishing” (scoring at least one of the three hedonic well-being symptoms (items 1–3) and at least 6 of the 11 positive functioning symptoms (items 4–14) with “every day” or “almost every day”); (b) “languishing” (scoring at least one of the three hedonic well-being symptoms and at least 6 of the 11 positive functioning symptoms with “never” or “once or twice”); or (c) “moderate mental health” (neither “languishing” nor “flourishing”). Moderate mental health and languishing were categorised as poor mental health. Participants were also asked about previous mental health diagnosis and whether they believed they need professional help” for mental health problems.

#### Statistical analysis

Statistical analysis was conducted in SPSS v27. Chi-squared tests were used to indicate whether there were differences in the distribution of demographic characteristics between participants in Australia or overseas. As many demographics contained multiple responses (e.g., education), chi-squared tests were conducted on merged groups (e.g., bachelor’s degree or above). These comparison groups are listed per demographic in Table 1; 95% confidence intervals (95% CI) were used to compare group differences across psychological distress, perceived stress, and mental wellbeing. Non overlapping 95% CIs were interpreted as a statistically significant difference (*p* ≤ .01) between two independent means [21]. We note that even overlapping CI may relate to statistically significant (*p* < .05) differences between two independent means when both samples are large enough (i.e., > 10) and the margins of error (i.e., width of the CIs) do not differ by more than a factor of 2 [21], however, we chose to focus on non-overlapping CIs for the ease of interpretation. Information from the text box which provided participants the opportunity to elaborate on their experience was analysed qualitatively with a publication currently in preparation.

**Table 1:** Characteristics of 3968 survey participants currently in Australia or overseas

	In Australia	Abroad	χ^**2**^	Australian adults [17]
n	2516 (63.4%)	1452 (36.6%)		
**Age**				
Mean (sd)	38.3 (10.7)	36.5 (12.1)		
Age group (years)				
18-24 (%)	118 (4.7)	154 (10.6)	101.98 (5), *p* < .001	10.3%
25-34 (%)	983 (39.1)	655 (45.1)		18.8%
35-45 (%)	781 (31.0)	305 (21.0)		17.6%
45-54 (%)	361 (14.3)	159 (11.0)		17.3%
55-64 (%)	194 (7.7)	109 (7.5)		15.4%
65 or more (%)	53 (2.1)	47 (3.3)		20.5%
**Gender**				
Female (%)	1970 (78.3)	1111 (76.5)	1.75 (1), *p* = .186	50.7%
**Citizenship**				
Australian (%)	1585 (63.0)	744 (51.2)	48.21 (1), *p* < .001	
**State/Territory**				
New South Wales (%)	754 (30.0)	–	–	32.2%
Victoria (%)	577 (22.9)	–		24.9%
Queensland (%)	449 (17.8)	–		20.3%
Western Australia (%)	336 (13.4)	–		10.4%
South Australia (%)	211 (8.4)	–		7.3%
Australian Capital Territory (%)	98 (3.9)	–		1.6%
Northern Territory (%)	20 (0.8%)	–		1.0%
Tasmania (%)	17 (0.7%)	–		2.3%
**Place of birth**				
Born overseas	1827 (72.6)	806 (56.5)	131.74 (1), *p* < .001	33.3%
**Ethnicity**				
Caucasian/European (%)	1919 (76.3)	972 (66.9)	80.82 (2), *p* < .001	
Asian/Indian (%)	385 (15.3)	390 (26.9)	(Caucasian/ European vs Asian vs other)	
Other/mixed ethnicity (%)	73 (2.9)	32 (2.2)		
Hispanic /Latin American (%)	63 (2.5)	14 (1.0)		
African (%)	17 (0.7)	10 (0.7)		
Aboriginal/Torres Strait Islander(%)	12 (0.5)	1 (0.1)		
Prefer not to say (%)	43 (1.7)	29 (2.0)		
**Education**				
Bachelor degree and above (%)	1902 (75.6)	1104 (76.0)	0.19 (1), *p* < .67	22.0%
**Employment**				
Full-time (%)	1367 (54.3)	801 (55.2)	124.04 (1), *p* < .001	57.7%
Part-time (%)	517 (20.5)	160 (11.0)	(employed vs unemployed)	30.4%
Parental leave (%)	159 (6.3)	28 (1.9)		–
Retired (%)	85 (3.4)	70 (4.8)		–
Unemployed (%)	185 (7.4)	284 (19.6)		6.9%
Other (%)	201 (8.0)	107 (7.4)		–
**Annual household income**				
Less than $25,000 (%)	76 (3.0)	179 (12.3)	331.43 (5), *p* < .001	
$25,000 - $50,000 (%)	201 (8.0)	197 (13.6)		
$50,000 - $100,000 (%)	595 (23.6)	313 (21.6)		
$100,000 - $200,000 (%)	906 (36.0)	277 (19.1)		
More than $200,000 (%)	429 (17.1)	173 (11.9)		
No income (%)	32 (1.3)	88 (6.1)		
Prefer not to answer (%)	270 (10.7)	222 (15.3)		
**Marital status**				
Single (%)	226 (9.0%)	295 (20.3%)	126.85 (5), *p* < .001	–
In a relationship (%)	491 (19.5%)	327 (22.5%)		–
Married (%)	1213 (48.2%)	577 (39.7%)		48.1%
De facto (%)	467 (18.6%)	195 (13.4)		10.4%
Divorced/separated (%)	92 (3.7%)	38 (2.6%)		11.7%
Widowed (%)	14 (0.6%)	6 (0.4%)		5.2%
Prefer not to answer (%)	11 (0.4%)	12 (0.8%)		–
**Children** (%)	1358 (54.0)	448 (30.9)	195.81 (1), *p* < .001	
**Previously diagnosed with mental health issue** (%)	762 (30.3)	365 (25.1)	10.57 (1), *p* < .001	
**Believe need help for a mental health problem** (%)	478 (19.0)	288 (19.8)	0.02 (1), *p* <. 90	

#### Ethics

The study was approved by the Flinders University Human Research Ethics Committee (Project ID: 4534). All participants provided informed consent online before commencing the survey.

## Results

From 8151 individuals who showed interest in the study, 2297 only clicked on the link, 949 did not respond to required questions about the impact of the COVID-19 border closure, 776 did not pass the attention check, 62 did not complete the main outcome measure of psychological distress (K10), 17 did not consent, and 82 indicated that they were positively or “neither” affected. In total, 3968 participants indicated they were negatively affected by the international border closure and were included in the analysis. While the demographic characteristics of participants broadly reflected the Australian population, the proportion of younger adults and females was higher, while education was skewed towards higher levels. Participants overseas resided in 92 different countries, most commonly in the UK (432, 10.9%), India (145, 3.7%), USA (119, 3.0%), Germany (63, 1.6%) and United Arab Emirates (60, 1.5%), and were primarily of Caucasian/European (2891, 72.9%) or Asian/Indian ethnicity (775, 19.5%).

### Impact of the COVID-19 international border closure

#### Scenarios

From 2516 respondents in Australia, 1174 individuals (29.6% of total sample) wanted to leave Australia, while 1015 (25.6%) wanted someone to enter the country from overseas and 327 (8.2%) reported other reasons (e.g., 255 reported having been affected by both). From the respondents overseas, 1223 (30.8%) wanted to enter Australia and 111 individuals wanted someone to leave Australia (2.8%) and 118 (3.0%) reported other reasons (e.g., 71 reported having been affected by both) (Table 2).

**Table 2:** Levels of psychological distress, perceived stress and wellbeing by scenario

Scenario	Wanting to leave Australia	Wanting someone else to enter Australia	Wanting to enter Australia	Wanting someone else to leave Australia
n	1174 (29.6%)	1015 (25.6%)	1223 (30.8%)	111 (2.8%)
**Psychological distress**				
Mean (sd)	29.5 (8.4)	31.3 (8.4)	31.1 (8.9)	29.1 (8.2)
[95%CI]	[29.0; 29.9]	[30.7; 31.8]	[30.6; 31.6]	[27.5; 30.6]
Low distress (%)	62 (5.3%)	31 (3.1%)	53 (4.3%)	4 (3.6%)
Moderate distress (%)	153 (13.0%)	102 (10.0%)	140 (11.4%)	16 (14.4%)
High distress (%)	354 (30.2%)	282 (27.8%)	324 (26.5%)	45 (40.5%)
Very high distress (%)	605 (51.5%)	600 (59.1%)	706 (57.7%)	46 (41.4%)
**Perceived Stress**				
Perceived stress, mean (sd)	12.6 (4.1)	13.3 (4.1)	13.0 (4.3)	12.3 (4.5)
[95% CI]	[12.4; 12.8]	[13.0; 13.5]	[12.7; 13.2]	[11.4; 13.2]
Perceived control, mean (sd)	6.4 (2.2)	6.6 (2.2)	6.6 (2.3)	6.1 (1.9)
[95% CI]	[6.2; 6.5]	[6.4; 6.7]	[6.5; 6.8]	[5.7; 6.4]
**Mental wellbeing**				
Mean (sd)	29.8 (15.0)	30.3 (14.0)	29.5 (15.3)	34.3 (13.9)
[95% CI]	[28.9; 30.7]	[29.4; 31.2]	[28.5; 30.5]	[31.4; 37.1]

Note. Not included in Table: *n* = 327 (8.2%) of those in Australia reported “other”; *n* = 118 (3.0%) of those overseas reported “other”; *n* = 337 (8.5%) did not complete questions about perceived stress; *n* = 583 (14.7%) did not complete questions about mental wellbeing

#### Reasons

Independent of location, 3248 respondents reported separation from their partner, family, and friends as the main reason for wanting to enter or leave the country (3248, 81.1%; 2223, 88.4% in Australia; and 1025 70.6% overseas). Of those overseas, 163 respondents reported study (4.1%), and 194 reported employment (4.9%) as other main reasons for entering Australia (Table 3). Irrespective of the reason why they had been affected, respondents perceived a high negative impact on their mental health (visual analogue scale of 0-100) with interruption to study being considered to have the greatest impact on mental health (91.1, 95% CI, 88.9 – 93.2), and interruptions to holiday plans the lowest (48.5, 95% CI, 40.0 – 57.1).

**Table 3:** Levels of psychological distress, perceived stress and wellbeing by reason

Reason	To be with partner, family, friends	Employment or economic reasons	Study^**a**^	Holiday plans	Personal safety and health	Other
**Total** (%)	3248 (81.1)	194 (4.9)	163 (4.1)	54 (1.4)	104 (2.6)	129 (3.3)
**In Australia** (%)	2223 (88.4)	68 (2.7)	8 (0.3)	48 (1.9)	71 (2.8)	45 (1.8)
**Overseas** (%)	1025 (70.6)	126 (8.7)	155 (10.7)	6 (0.4)	33 (2.3)	84 (5.8)
**Negative effect**						
Mean (sd)	87.7 (15.1)	85.3 (17.3)	91.1 (13.7)	48.5 (30.1)	90.1 (14.8)	86.4 (16.3)
[95%CI]	[87.2; 88.3]	[82.8; 87.8]	[88.9; 93.2]	[40.0; 57.1]	[87.2; 93.2]	[83.5; 89.3]
**Psychological distress**						
Mean (sd)	30.2 (8.4)	31.1 (8.8)	35.7 (8.2)	21.9 (9.2)	33.7 (7.7)	31.8 (9.5)
[95%]	[29.9; 30.4]	[29.8; 32.3]	[34.4; 37.0]	[19.3; 24.4]	[32.2; 35.2]	[30.1; 33.4]
Low distress (%)	134 (4.1)	8 (4.1)	2 (1.2)	17 (31.5)	1 (1.0)	5 (3.9)
Moderate distress (%)	402 (12.4)	20 (10.3)	10 (6.1)	10 (18.5)	6 (5.8)	18 (14.0)
High distress (%)	955 (29.4)	55 (28.4)	25 (15.3)	16 (29.6)	17 (16.3)	31 (24.0)
Very high distress (%)	1757 (54.1)	111 (57.2)	126 (77.3)	11 (20.4)	80 (76.9)	75 (58.1)
**Perceived Stress**						
Perceived stress, mean (sd)	12.9 (4.1)	12.4 (4.3)	14.2 (4.0)	8.6 (5.5)	14.4 (3.4)	13.2 (4.5)
[95% CI]	[12.8; 13.1]	[11.8; 13.1]	[13.5; 14.9]	[7.0; 10.1]	[13.7; 15.1]	[12.4; 14.0]
Perceived control, mean (sd)	6.5 (2.1)	6.5 (2.4)	6.9 (2.7)	4.7 (3.0)	6.7 (2.3)	6.8 (2.6)
[95% CI]	[6.5; 6.6]	[6.1; 6.8]	[6.4; 7.4]	[3.9; 5.5]	[6.3; 7.2]	[6.3; 7.2]
**Mental wellbeing**						
Mean (sd)	30.3 (14.4)	29.8 (16.0)	24.3 (15.2)	42.9 (17.9)	25.9 (13.8)	29.8 (17.2)
[95% CI]	[28.8; 30.9]	[27.2; 32.3]	[21.5; 27.0]	[37.7; 48.1]	[22.9; 28.9]	[26.6; 33.1]

Note. *n* = 76 (1.9%) did not answer the question about reason; *n* = 337 (8.5%) did not complete questions about perceived stress; *n* = 583 (14.7%) did not complete questions about mental wellbeing. ^a^ *n* = 137 (84% of those who reported study as the main reason) indicated international student status

### Mental health and wellbeing

On average the sample reported very high levels of psychological distress and perceived stress as well as low levels of mental wellbeing (Table 4). Overall, 3316 participants (83.6%) reported high (28.4%) or very high levels of distress (55.2%) (Table 4). There was a small but significant difference between Australian citizens (29.3, 95% CI, 28.9 – 29.6) or permanent resident (30.0, 95% CI, 29.4 – 30.6) compared with those holding an Australian visa (33.7, 95% CI, 33.1 – 34.4) (Supplementary data, Table S1). Levels of perceived stress and mental wellbeing followed a similar pattern (Table 4).

**Table 4:** Levels of psychological distress and wellbeing in the total sample (in Australia and overseas)

	Total sample	In Australia	Overseas
n	3968	2516 (63.4%)	1452 (36.6%)
**Psychological distress**			
Mean (sd)	30.4 (8.6)	30.1 (8.5)	30.9 (8.7)
[95%CI]	[30.1; 30.7]	[29.8; 30.5]	[30.4; 31.3]
Low distress (%)	176 (4.0%)	116 (4.6%)	60 (4.1%)
Moderate distress (%)	476 (12.0%)	305 (12.1%)	171 (11.8%)
High distress (%)	1126 (28.4%)	728 (28.9%)	398 (27.4%)
Very high distress (%)	2190 (55.2%)	1367 (54.3%)	823 (56.7%)
**Perceived Stress**			
Perceived stress, mean (sd)	12.9 (4.2)	12.9 (4.1)	12.9 (4.2)
[95% CI]	[12.8; 13.0]	[12.7; 13.1]	[12.7; 13.2]
Perceived control, mean (sd)	6.5 (2.2)	6.5 (2.2)	6.6 (2.3)
[95% CI]	[6.5; 6.6]	[6.4; 6.6]	[6.5; 6.7]
**Mental wellbeing**			
Mean (sd)	30.2 (14.8)	30.4 (14.7)	30.0 (15.1)
[95% CI]	[29.7; 30.7]	[29.7; 31.0]	[29.1; 30.9]
Flourishing (%)	498 (12.6%)	329 (13.1%)	169 (11.6%)
Moderate mental health (%)	1932 (48.7%)	1276 (50.7%)	656 (45.2%)
Languishing (%)	1036 (26.1%)	664 (26.4%)	372 (25.6%)

Note. *n* = 337 (8.5%) did not complete questions about perceived stress; *n* = 583 (14.7%) did not complete questions about mental wellbeing

#### Scenarios

Differences in psychological distress were observed across scenarios: among participants overseas those who wanted to enter Australia (31.1, 95% CI, 30.6 – 31.6) or among participants residing in Australia those who wanted someone else to come to Australia (31.3, 95% CI, 30.7 – 31.8) reported higher distress compared those who wanted to leave (29.4, 95% CI 29.0 – 29.9) or those who wanted someone else to leave Australia (29.1, 95% CI, 27.5 – 30.6), respectively. Similar patterns were observed in perceived stress and mental wellbeing (Table 2).

#### Reasons

Those who reported study (35.7, 95% CI, 34.4 – 37.0), of which 84% were international students, and personal safety (33.7, 95%, 32.2 – 35.2) as reasons to enter or leave the country, reported the highest distress, followed by those who reported employment/economic reasons (31.1, 95% CI, 29.8 – 32.3) and separation from partner, family, and friends (30.1, 95% CI, 29.9 – 30.4). However, those who specifically reported being separated from their partner (34.0, 95% CI, 33.5 – 34.5) reported distress levels similar to those affected by study or personal safety. Those who reported holiday as the main reason showed the lowest level of psychological distress (21.9, 95% CI, 19.3 – 24.4). Again, similar patterns were observed in perceived stress and mental wellbeing (Table 3).

## Discussion

We found very high levels of distress and perceived stress as well as low wellbeing among individuals who reported being negatively affected by the current COVID-19 international border closure. Almost two thirds of the respondents (63.4%) in our study were residing in Australia, while the remaining resided in over 90 different countries. The three most common scenarios whereby participants reported being negatively affected included wanting to enter (30.8%) or leave Australia (29.6%) or wanting someone else to enter Australia (25.6%). The majority of participants who reported being negatively affected (81.1%) wanted to be with a family member (e.g., partner, parents, children etc.), while a smaller number cited employment and economic factors, study, health, and safety as well as holiday plans as reasons. This data demonstrates the diversity of scenarios and different reasons as to how and why individuals are currently impacted by the international border closure.

Regardless of participants’ location and independent of the specific scenario or reason why individuals were affected, we found that mean levels of psychological distress and perceived stress were very high, while wellbeing was low among individuals who reported having been negatively affected by the current COVID-19 Australian international border closure. While the current sample broadly reflected the demographic characteristics of the Australian population, participants’ level of income and employment rates were higher suggesting that they were not experiencing COVID-19-related financial distress, a factor that was associated with higher psychological distress previously [5]. Despite this, the data shows that mean levels of distress were substantially higher than population data from Australia prior to [22] and, more importantly, during the time of the COVID-19 pandemic [7, 17]. Levels of psychological distress (83.6% K10 > 22) were substantially higher than recent surveys during the COVID-19 pandemic reported from individuals in lockdown (30.3% K10 > 12) [23], quarantine (7.1% K10 > 22) [24], and other vulnerable groups including healthcare workers (46% K10 > 22) [25]. Reported levels of perceived distress were also substantially higher than those found in previous studies in Australia [26] while results for wellbeing followed a similar pattern. These results are in line with findings from a recent scoping review demonstrating the unintended harmful negative consequences on the wellbeing and mental health of those affected by border restrictions [11].

While respondents who wanted to enter Australia to study, those who were separated from their partner, those who wanted to enter or leave the country due to health or personal safety, and visa holders reported the highest magnitude of negative mental health outcomes, it is important to note that mental health and wellbeing was poor across the different scenarios and reasons. These findings are not surprising for several reasons. At the beginning of the pandemic, adults in Australia ranked the health and wellbeing of family and loved ones as their three primary concerns [27]. Not being able to be with loved ones and have access to social support during unprecedented times of a pandemic has been identified as being extremely challenging and results in psychological distress [28]. In a similar vein, having uncertainty about study, employment or personal health and safety likely increases anxiety about the future [29]. Particularly, international students have been highlighted as a vulnerable group during the pandemic with research demonstrating the high prevalence of mental health problems, including symptoms of depression, anxiety and stress [30]. The very high levels of psychological distress across these samples contrast with the small group of participants citing disruptions to their holiday plans as the least affected group (50.0% K10 > 22).

### Strengths and limitations

This is the first study investigating the mental health of individuals reporting being negatively affected by the current international border closure in Australia using standardised psychometric measures which allow for comparison with other populations. Respondents comprised a large sample of individuals currently in Australia as well as overseas who reported being negatively affected by the current COVID-19 international border closure. However, as participation in the study was self-selected and individuals were mostly recruited via Facebook targeting affected groups (e.g., individuals overseas who are attempting to return to Australia), the representativeness of the sample is limited. While we tried to minimise any potential bias by asking respondents whether they had been positively or negatively affected, only 81 respondents reported a positive or “neutral” affect suggesting that the survey attracted predominantly those who were negatively affected. It is likely that overall levels of distress would have been much lower if the study had included a representative sample that include those who were positively or neutrally affected by the international border closure. Second, due to the online nature of our recruitment and assessment, we may not have captured individuals without internet access or lower computer proficiency. Third, given the nature of such surveys and the likely multicultural nature of the sample, there is an inherent bias towards English speakers. Furthermore, due to the cross-sectional nature of the survey, it is not possible to draw any causal conclusions. While it is feasible that the current psychological distress is closely associated with the current border closure, other factors or past experiences may have additional or cumulative impact. It is therefore necessary to investigate the longer-term mental health implications among affected individuals and to examine more nuanced aspects of the experiences of those in each of the scenarios identified here.

### Implications

Without implying causation between the restrictions in place for international travel from or to Australia and high levels of psychological distress, it is important clinically and for policy purposes to characterise the negative effects associated with the border closure. It is equally important in future studies to track the mental health of these groups. If the negative mental health profile reported here deteriorates over time, then it is very likely that services and clinicians will need to respond more emphatically and that policy makers will need to consider how to nuance the border closure policy in order to mitigate the mental health risks.

Findings from the current study suggest the importance of assessing mental health symptoms in primary care among those who have been born overseas or have family (especially partners) who are currently overseas. It might also be helpful to guide these individuals to existing primary care and mental health services, inclusive of digital resources, and encourage them to make their mental health and wellbeing a priority as external factors may not change in the near future. Clinicians need to help affected individuals develop problem-solving and coping strategies to manage distress. Given the high magnitude of distress and symptoms, consideration needs to be given as to whether affected individuals are experiencing a mental health disorder and what appropriate responses are viable. Some consideration of the language and cultural obstacles to the use of existing resources will need to be made. It is also critical to consider how best to support the mental health and wellbeing of Australian citizens and permanent residents who are currently overseas attempting to return to Australia. This seems particularly important as current Australian mental health support organisations such as for example, Beyond Blue and Lifeline, do not offer support to those who are currently overseas.

## Conclusions

We found substantially higher levels of psychological distress among individuals who reported having been negatively affected by the COVID-19 international border closure compared to other vulnerable groups during the pandemic such as individuals in lockdown [23], quarantine [24] or healthcare workers [25]. Our findings indicate that respondents were similarly affected, whether they were in Australia or overseas. Health and mental health care providers should be aware of this crisis and provide appropriate support options and practical strategies to mitigate the risk of further deterioration. Policy decisions need to take the significant mental health cost of these border restrictions into consideration when developing future strategies and plans.

## Supplementary Information

**Additional file 1: Supplementary Table.** Levels of mental health and wellbeing by Australian citizenship status.

## Data Availability

The datasets generated and analysed during the current study are available from the corresponding author on reasonable request.

## Abbreviations

COVID-19: Coronavirus Disease 2019
K10: Kessler Psychological Distress Scale
PSS: The Perceived Stress Scale
MHC-SF: Mental Health Continuum Short Form
SPSS: IBM Statistical Package for Social Science
CI: Confidence Interval
SD: Standard Deviation

## References

[1] Aknin LB, De Neve JE, Dunn EW, Fancourt D, Goldberg E, Helliwell J (2021). Mental health during the first year of the COVID-19 pandemic: A Review and Recommendations for Moving Forward.

[2] McGorry P (2020). Mental health and COVID-19: are we really all in this together?. Med J Aust.

[3] Stanaway F, Irwig LM, Teixeira-Pinto A, Bell KJ (2021). COVID-19: estimated number of deaths if Australia had experienced a similar outbreak to England and Wales. Med J Aust.

[4] Iasiello M, van Agteren J, Muir CE (2020). Mental Health and/or Mental Illness: A Scoping Review of the Evidence and Implications of the Dual-Continua Model of Mental Health: Exeley Inc.

[5] Batterham PJ, Calear AL, McCallum SM, Morse AR, Banfield M, Farrer LM (2021). Trajectories of depression and anxiety symptoms during the COVID-19 pandemic in a representative Australian adult cohort. Med J Aust.

[6] Fisher JR, Tran TD, Hammarberg K, Sastry J, Nguyen H, Rowe H (2020). Mental health of people in Australia in the first month of COVID-19 restrictions: a national survey. Med J Aust.

[7] Rahman MA, Hoque N, Alif SM, Salehin M, Islam SMS, Banik B (2020). Factors associated with psychological distress, fear and coping strategies during the COVID-19 pandemic in Australia. Glob Health.

[8] Chiesa V, Antony G, Wismar M, Rechel B. COVID-19 pandemic: health impact of staying at home, social distancing and ‘lockdown’ measures—a systematic review of systematic reviews. J Public Health. 2021. 10.1093/pubmed/fdab102 PMC8083256 33855434

[9] Bao L, Li W-T, Zhong B-L (2021). Feelings of loneliness and mental health needs and services utilization among Chinese residents during the COVID-19 epidemic. Glob Health.

[10] Prati G, Mancini AD (2021). The psychological impact of COVID-19 pandemic lockdowns: a review and meta-analysis of longitudinal studies and natural experiments. Psychol Med.

[11] Klinger C, Burns J, Movsisyan A, Biallas R, Norris SL, Rabe JE, et al. Unintended health and societal consequences of international travel measures during the COVID-19 pandemic: a scoping review. J Travel Med. 2021;28(7):taab123. 10.1093/jtm/taab123. 10.1093/jtm/taab123 PMC8436381 34369562

[12] Adekunle A, Meehan M, Rojas-Alvarez D, Trauer J, McBryde E (2020). Delaying the COVID-19 epidemic in Australia: evaluating the effectiveness of international travel bans. Aust N Z J Public Health.

[13] Department of Home Affairs. Travel restrictions and exemptions 2021 [25 August 2021]. Available from: https://covid19.homeaffairs.gov.au/travel-restrictions.

[14] Australian Government Department of Infrastructure, Transport, Regional Development and Communications. International air passenger arrival caps 2021 [25 August 2021]. Available from: https://www.infrastructure.gov.au/department/media/mr_20210825-int-air-passenger-arrival-caps.aspx.

[15] Australian Government Department of Home Affairs. Freedom of Information Request FA 20/09/00219 2020 [25 August 2021]. Available from: https://www.homeaffairs.gov.au/foi/files/2020/fa-200900219-document-released.PDF.

[16] United Nation World Tourism Organization. Vaccines and Digital Solutions to Ease Travel Restrictions 2021 [25 August 2021]. Available from: https://www.unwto.org/news/vaccines-and-digital-solutions-to-ease-travel-restrictions.

[17] Australian Bureau of Statistics (2016). 2016 Census QuickStats.

[18] Kessler RC, Andrews G, Colpe LJ, Hiripi E, Mroczek DK, Normand SL (2002). Short screening scales to monitor population prevalences and trends in non-specific psychological distress. Psychol Med.

[19] Ribeiro Santiago PH, Nielsen T, Smithers LG, Roberts R, Jamieson L (2020). Measuring stress in Australia: validation of the perceived stress scale (PSS-14) in a national sample. Health Qual Life Outcomes.

[20] Keyes CL (2005). Mental illness and/or mental health? Investigating axioms of the complete state model of health. J Consult Clin Psychol.

[21] Cumming G, Finch S (2005). Inference by eye: confidence intervals and how to read pictures of data. Am Psychol.

[22] Slade T, Grove R, Burgess P (2011). Kessler psychological distress scale: normative data from the 2007 Australian National Survey of mental health and wellbeing. Aust N Z J Psychiatry.

[23] Every-Palmer S, Jenkins M, Gendall P, Hoek J, Beaglehole B, Bell C (2020). Psychological distress, anxiety, family violence, suicidality, and wellbeing in New Zealand during the COVID-19 lockdown: a cross-sectional study. PLoS One.

[24] D'Onise K, Meena S, Venugopal K, Currie M, Kirkpatrick E, Hurley J (2021). Holistic approach supporting mental wellbeing of people in enforced quarantine in South Australia during the COVID-19 pandemic. Aust N Z J Public Health.

[25] Adams MA, Brazel M, Thomson R, Lake H. The mental health of Australian medical practitioners during Covid-19. Australas Psychiatry. 2021:10398562211010807. 10.1177/10398562211010807 34010578

[26] Australian Psychological Society (2015). 2015 Stress and Wellbeing Survey.

[27] Rossell SL, Neill E, Phillipou A, Tan EJ, Toh WL, Van Rheenen TE (2021). An overview of current mental health in the general population of Australia during the COVID-19 pandemic: results from the COLLATE project. Psychiatry Res.

[28] Nitschke JP, Forbes PAG, Ali N, Cutler J, Apps MAJ, Lockwood PL (2021). Resilience during uncertainty? Greater social connectedness during COVID-19 lockdown is associated with reduced distress and fatigue. Br J Health Psychol.

[29] Rettie H, Daniels J. Coping and tolerance of uncertainty: predictors and mediators of mental health during the COVID-19 pandemic. Am Psychol. 2021;76(3):427–37. 10.1037/amp0000710. 10.1037/amp0000710 32744841

[30] Alam MD, Lu J, Ni L, Hu S, Xu Y. Psychological outcomes and associated factors among the international students living in China during the COVID-19 pandemic. Front Psychiatry. 2021;12(1372). 10.3389/fpsyt.2021.707342 PMC8414650 34483997

