# Supplementary material for: A cross-sectional investigation of the mental health and wellbeing among individuals who have been negatively impacted by the COVID-19 international border closure in Australia
Source: Global Health. 2022 Feb 8;18:12. doi: 10.1186/s12992-022-00807-7 (PMC8822815; doi:10.1186/s12992-022-00807-7)
Supplement: Supplementary file 1 — Additional file 1: Supplementary Table. Levels of mental health and wellbeing by Australian citizenship status. [file 12992_2022_807_MOESM1_ESM.docx]

Supplementary Table. Levels of mental health and wellbeing by Australian citizenship status

| **Citizenship status** | **Australian citizen** | **Permanent resident** | **Visa holder** | **Other** |
| --- | --- | --- | --- | --- |
| n | 2,329 | 664 | 672 | 280 |
| **Psychological distress**  Mean (sd)  [95% CI]  Low distress (%)  Moderate distress (%)  High distress (%)  Very high distress (%) | 29.3 (8.6)  [28.9, 29.6]  136 (5.8%)  315 (13.5%)  712 (30.6%)  1,166 (50.1%) | 30.0 (8.0)  [29.4, 30.6]  21 (3.2%)  93 (14.0%)  190 (28.6%)  360 (54.2%) | 33.7 (8.3)  [33.1, 34.4]  13 (1.9%)  45 (6.7%)  142 (21.1%)  472 (70.2%) | 32.8 (8.2)  [31.8, 33.8]  5 (1.8%)  20 (7.1%)  75 (26.8%)  180 (64.3%) |
| **Perceived Stress**  Perceived stress, mean (sd)  [95% CI]  Perceived control, mean (sd)  [95% CI] | 12.6 (4.2)  [12.4, 12.8]  6.4 (2.2)  [6.3, 6.5] | 13.0 (3.9)  [12.7, 13.3]  6.6 (2.0)  [6.5, 6.8] | 13.8 (4.1)  [13.5, 14.1]  6.8 (2.4)  [6.6, 7.0] | 13.4 (4.1)  [12.9, 13.9]  6.9 (2.0)  [6.7, 7.1] |
| Mental wellbeing  Mean (sd)  [95% CI] | 31.4 (15.0) [30.8, 32.1] | 29.2 (13.6) [28.1, 30.4] | 27.2 (15.4) [25.8, 28.5] | 28.5 (14.1) [26.7, 30.4] |

Note. n = 23 (0.6%) did not complete question about Australian citizenship status; n = 337 (8.5%) did not complete questions about perceived stress; n = 583 (14.7%) did not complete questions about mental wellbeing.
